# Effect of atrazine and curcumin on mouse I-10 Leydig cells: steroidogenesis and beyond

**DOI:** 10.3389/fendo.2026.1866034

**Published:** 2026-08-14

**Authors:** Hoda Elkady, Pengli Bu

**Affiliations:** Department of Pharmaceutical Sciences, College of Pharmacy and Health Sciences, St. John’s University, Queens, NY, United States

**Keywords:** atrazine, curcumin, Leydig cell, steroidogenesis, testosterone

## Abstract

**Introduction:**

Chronic exposure to pesticides is increasingly becoming a concern in the modern world. Atrazine (ATZ), a widely used pesticide in the U.S., is known to interfere with the male reproductive system. In the present study, we examined the effects of ATZ on viability, cell cycle progression, steroid production, and steroidogenic gene expression profile of testicular Leydig cells using the mouse Leydig cell line I-10 as an *in vitro* model. We then determined whether curcumin (Cur), an herbal medicine, can counteract the effects of ATZ. Molecular docking was performed to assess the potential of direct binding of ATZ and Cur to key steroidogenic factors of both human and mouse origins.

**Methods:**

I-10 cells were treated for 6 days with either ATZ (3.3, 10, 33, 100 µM), Cur (3, 10, 30 µM), or a co-treatment of Cur (3 µM) + ATZ (33 or 100 µM). Cell viability, cell cycle progression, production of progesterone (P4) and testosterone (T), and expression of key steroidogenic genes were assessed on Days 2, 4, and 6. Additionally, AutoDock Vina v1.2.7 was used to predict the binding affinities of Cur and ATZ to key mouse and human steroidogenic proteins in comparison to their endogenous substrates.

**Results:**

ATZ decreased I-10 cell viability while increasing T levels in a concentration-dependent manner. Cur transiently stimulated I-10 cell proliferation and increased P4 but showed mixed effects on T levels. Co-treatment decreased viability, as supported by changes in cell cycle distributions. Co-treatment also significantly increased both P4 and T production, with induction of expression of the steroidogenic genes StAR, Cyp11a1, Hsd3β6, and Hsd17β3 at specific time points. Furthermore, Cur exhibited strong predicted binding to both mouse and human steroidogenic proteins, comparable to that of their endogenous substrates.

**Conclusion:**

Our findings suggest a potential interaction between Cur and ATZ, which was more prominent at ATZ 100 μM, stimulating steroidogenesis in I-10 Leydig cells at the expense of decreased viability.

## 1 Introduction

Testosterone (T) is the principal male sex hormone necessary for overall well-being in men (1). Decreases in circulating T levels are associated with inescapable aspects of life – the aging process, disease, medication, and exposure to environmental contaminants. These stressors, whether natural or man-made, contribute to the decline of T, particularly in older men. Reduced T level, below the threshold needed for physiological homeostasis, is clinically referred to as male hypogonadism, dubbed “andropause” for middle-aged men suffering from hypogonadism (2, 3). Hypogonadism is typically manifested as reduced muscle growth, increased fat accumulation, low libido and energy levels, depressed mood, and decreased cognitive function (4–7). Therefore, it is of significant relevance to public health that we understand how these stressors contribute to T decline and then develop interventions to counteract or mitigate their negative impacts on T levels.

**Figure 1:**
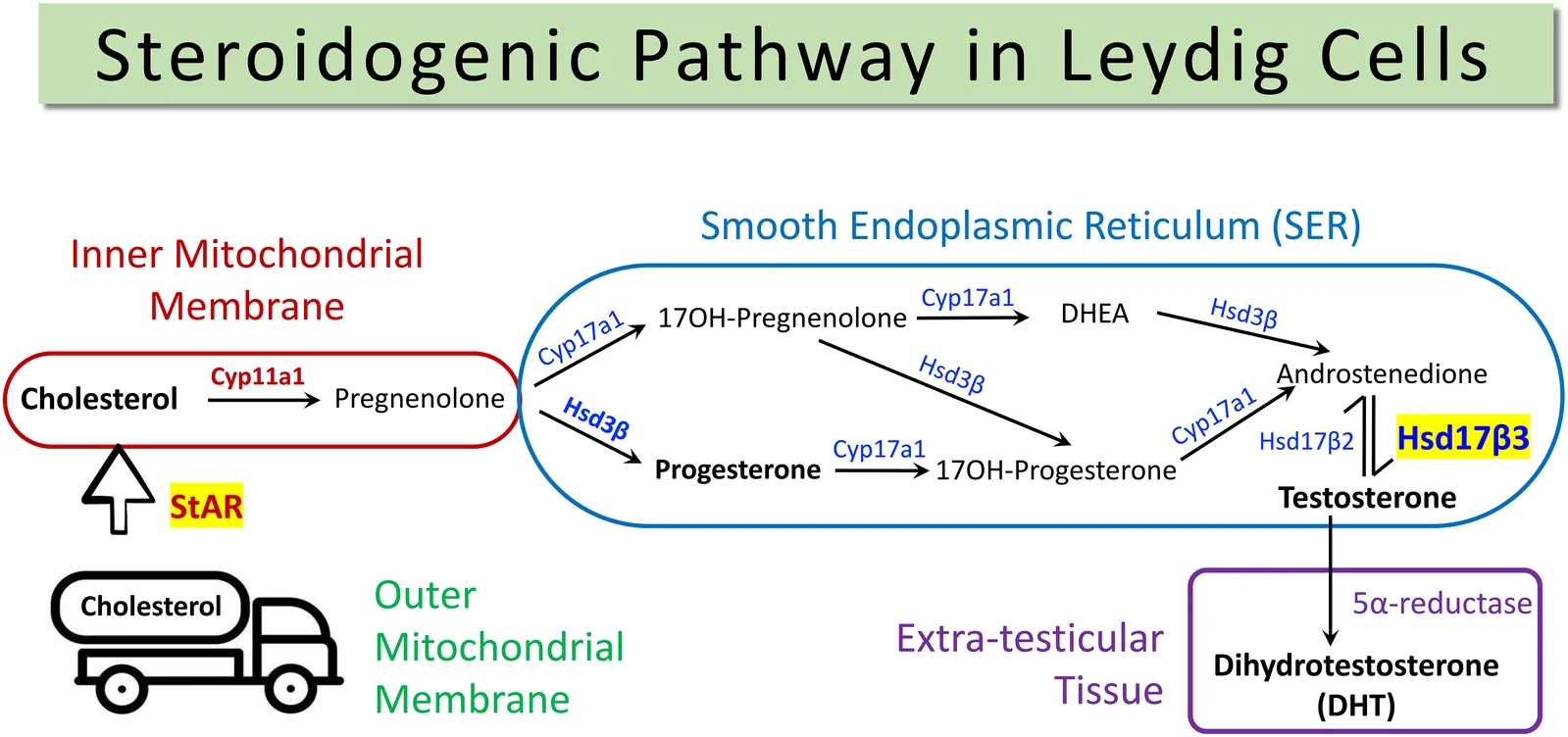
The established steroidogenic pathway in Leydig cells. Cholesterol is transported to the mitochondrial inner membrane by StAR and converted to pregnenolone by Cyp11a1, followed by a cascade of enzymatic reactions in the smooth endoplasmic reticulum to be converted to testosterone. In this study, the RNA levels of StAR, Cyp11a1, Hsd3β6, and Hsd17β3 were measured, along with the quantification of steroids progesterone (P4) and testosterone (T).

The majority of T is produced in a process called testicular steroidogenesis, occurring in the testicular Leydig cells (**Figure 1**). Testicular production of T accounts for approximately 95% of total T in males. Within the male body, the process of T production is driven by the Hypothalamic-Pituitary-Gonad (Testis) endocrine axis, which regulates T production at the organismal level (8). Although the decline in the Hypothalamic-Pituitary-Gonad (Testis) axis can cause androgen deficiency (9), the root cause of androgen deficiency originates primarily from the Leydig cells in a two-dimensional manner: (1) a decrease in Leydig cell number (10) and/or (2) their reduced steroidogenic capacity. Under normal conditions, adult Leydig cells do not proliferate once formed after puberty (11). This underscores the urgent yet unmet need to protect and revitalize adult Leydig cells to help prevent late-onset hypogonadism. The current study specifically aims to address this important issue.

The process by which T is produced and released by Leydig cells is summarized in **Figure 1**. Within Leydig cells, cholesterol in the outer mitochondrial membrane is transported into the inner mitochondrial membrane via the transporter protein steroidogenic acute regulatory protein (StAR), initiating T biosynthesis (12). In the inner mitochondrial membrane, cholesterol is hydroxylated and cleaved by Cyp11a1, an enzyme also known as the cytochrome P450 side-chain cleavage enzyme, to produce pregnenolone (13, 14). The transport of cholesterol via StAR and its subsequent conversion to pregnenolone by Cyp11a1 are both considered the rate-limiting steps in this pathway (15, 16). From there onward, pregnenolone passively diffuses out of the mitochondria into the smooth endoplasmic reticulum (SER), where it undergoes a cascade of enzymatic reactions catalyzed by various steroidogenic enzymes and is eventually converted to T (17). In the present study, we assessed the levels of both T and progesterone (P4), an intermediate precursor of T (18), as benchmark measures of steroidogenesis in Leydig cells. We attempted to connect changes in secreted levels of P4 and/or T to steroidogenic gene expression profiles, including the expression of Hsd3β6, a key enzyme involved in P4 production, and Hsd17β3, the enzyme needed for the conversion of androstenedione to T (19), in addition to StAR and Cyp11a1.

In the present study, we investigated the protective potential of curcumin (Cur), a natural herbal medicine derived from turmeric, either alone or in the presence of atrazine (ATZ), a common environmental contaminant, in I-10 Leydig cells. ATZ is one of the most widely used pesticides in the United States and China, primarily applied to corn, sorghum, and sugarcane (20). Although ATZ is a cost-effective herbicide, its widespread use is associated with several issues (21). Most notably, ATZ possesses endocrine-disrupting properties, has a relatively long half-life, and persists in soil, surface water, and groundwater (22). With the U.S. Environmental Protection Agency (EPA) loosening the ATZ concentration-equivalent level of concern (CE-LOC) in surface waters from 3.4 to 9.7 µg/L in 2024 (23), it is becoming increasingly necessary and urgent to understand how ATZ disrupts endocrine functions in general, and in particular, how it affects Leydig cells and their steroidogenic capacity.

Cur is known for its antioxidant, anti-inflammatory, and antimicrobial properties (24). For thousands of years, especially in Asian countries, Cur has been used in the form of turmeric as a natural herbal remedy to treat a variety of ailments (25). Despite its long-standing use in history and mounting preclinical data showing the various biological pathways it may act on (26), Cur is not currently approved clinically, primarily because of its low oral bioavailability (27). The key physicochemical properties of ATZ and Cur are listed in **Table 1**.

**Table 1:** Physicochemical properties of ATZ and Cur (28).

	Structure	Physical properties
ATZ	**Formula:** C_8_H_14_ClN_5_ 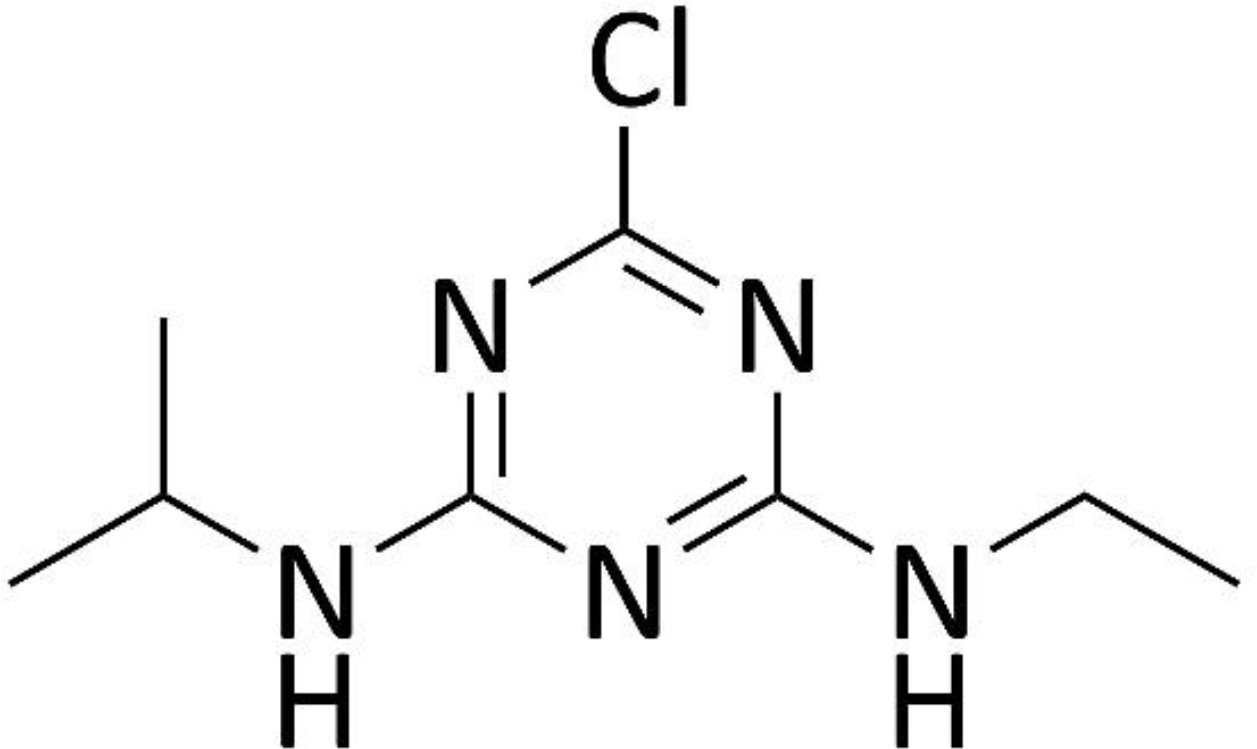	**Molecular Weight:** 215.68 g/mol **Number of Rotatable Bonds:** 4 **Number of Hydrogen Bond Acceptors:** 5 **Number of Hydrogen Bond Donors:** 2 **Polar Surface Area:** 62.7 Å²
Cur	**Formula:** C_21_H_20_O_6_break/> 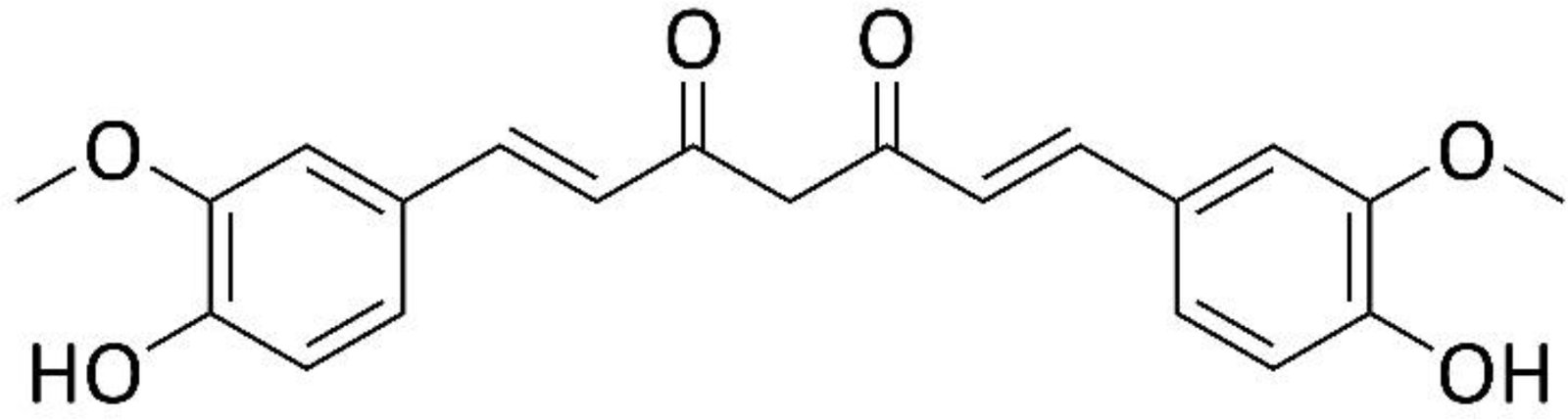	**Molecular Weight:** 368.4 g/mol **Number of Rotatable Bonds:** 8 **Number of Hydrogen Bond Acceptors:** 6 **Number of Hydrogen Bond Donors:** 2 **Polar Surface Area:** 93.1 Å²

Recently, we proposed Cur as a mechanism-based antidote to ATZ toxicity in the context of estrogen signaling with implications for breast cancer (28). However, its effects on the male reproductive system, particularly on T production, are less well-studied. In this study, we investigated the effect of Cur, alone and in the presence of ATZ, on viability, cell cycle progression, steroid production, and gene expression profiles of key steroidogenic enzymes, using the mouse Leydig tumor cell line I-10 as the *in vitro* model. Additionally, we have employed molecular docking experiments to predict direct binding of ATZ or Cur to the key steroidogenic transporter and enzymes.

## 2 Materials and methods

### 2.1 Chemicals

Atrazine (Cat. # HY-N7091, MedChemExpress; Purity: >98%) and curcumin (Cat. # C23025G, Tokyo Chemical Industry; Purity: >97%) were purchased from Fisher Scientific (Hanover Park, IL). Dimethylsulfoxide (DMSO) was purchased from ATCC (Manassas, VA). Reagents for gene expression analysis including TRIzol Reagent (Cat. # 15596018), Random Hexamer Primer (Cat. # SO142), Oligo(dT)18 Primer (Cat. # SO132), dNTP (10 mM each, Cat. # 18427013), SuperScript II Reverse Transcriptase (Cat. # 18064014), and SYBR Select Master Mix (Cat. # 4472908) were purchased from Thermo Fisher Scientific (Waltham, MA). Testosterone ELISA Kit (Cat. # 582701) and Progesterone ELISA Kit (Cat. # 582601) were purchased from Cayman Chemical (Ann Arbor, MI).

### 2.2 Culture of Leydig cells and treatment

Tumor-derived mouse Leydig I-10 cells (Cat. # CCL-83, ATCC) were cultured in F-12K medium (Cat. # 21127022, ThermoFisher Scientific) supplemented with Fetal Bovine Serum (2.5%, Cat. # 35-010-CV, Corning), Horse Serum (15%, Cat. # 16050122, ThermoFisher Scientific), and 25 mM HEPES (Cat. # 15630106, Gibco). Cells were maintained at 37 °C in a humidified atmosphere of 5% CO_2_. Cells in the logarithmic growth phase, between passages 2 and 6, were seeded at a density of 2.5 x 10^5^ cells per T-25 flask for time- and concentration-response studies. Treatment started 16–24 hours after seeding. The I-10 cells in T-25 flasks were treated with either vehicle (0.1% DMSO), ATZ (3.3, 10, 33, and 100 µM), Cur (3, 10, and 30 µM), or a co-treatment of Cur + ATZ (Cur 3 + ATZ 33 µM and Cur 3 + ATZ 100 µM) for 2, 4, and 6 days (n=3–4 flasks/treatment/time point), with medium and chemical change every 2 days. Cells were treated in fresh medium containing the specified chemical(s) at the designated concentrations and duration. At the time of harvest, viable cell count was determined using a hemocytometer or a Countess II automated cell counter (Cat. # AMQAX1000, Thermo Fisher Scientific). Every sample (n=3–4 independent biological replicates) was counted twice.

### 2.3 RNA extraction and real-time quantitative PCR

Attached cells from each treatment group were rinsed with 0.05% Trypsin, 0.53 mM EDTA (Cat. # 25-052-CI, Corning) once and incubated with 0.05% Trypsin, 0.53 mM EDTA for 2–5 minutes at 37 °C to facilitate detachment of viable cells, followed by neutralization with complete growth medium. Viable cells were then collected via centrifugation and lysed in TRIzol reagent, and total RNA extracted (Cat. # 15596018, Thermo Fisher Scientific) according to the manufacturer’s protocol and as we previously described (29–32). RNA samples were quantified on a NanoDrop 2000 Spectrophotometer (Thermo Fisher Scientific; Waltham, MA), and only samples with an A260/A280 ratio between 1.8 and 2.0 were used for subsequent reverse transcription. Using a 1:1 mixture of Random Hexamer and Oligo(dT)18 Primers, and SuperScript II Reverse Transcriptase, first-strand cDNA was reversely transcribed from each RNA sample (n=3–4 independent biological replicates) and used as template in the following quantitative PCR (qPCR) analysis on a StepOnePlus™ Real-Time PCR System (Applied Biosystems; Foster City, CA) using the following procedure: 20 µL reaction with an initial denature of 95 °C for 2 minutes followed by 40 cycles of 95 °C, 15 seconds and 60 °C, 1 minute; and melt curve (95 °C for 30 seconds, 65 °C for 30 seconds, and 95 °C for 30 seconds for one cycle). The relative RNA level of each gene of interest was calculated using the delta-delta CT method, with Gapdh as the housekeeping gene. Unless otherwise specified, the RNA level of any given gene in Day 2 DMSO treatment was set as 1 (or 100%), and the RNA levels of the same gene in all other groups (of the same time points as well as of different time points) were expressed as relative folds (or percentage) of Day 2 DMSO. The sequences of qPCR primers used are listed in **Table 2**.

**Table 2:** The sequences of primers used in real-time qPCR analysis.

Gene	Forward primer	Reverse primer
mGapdh	TGTGAACGGATTTGGCCGTA	ACTGTGCCGTTGAATTTGCC
mStAR	GAGCGTGCGCTGTACCAAGC	TCTGCTCCGGCATCTCCCCA
mCyp11a1	CCCCTGGGTGGCCTATCACCA	ACCTTCCAGCAGGGGCACGA
mHsd3β6	ACCAGGTCCTCCAGTAAGGACAAAA	AGGCTTGTGGCTGGGTCAGTG
mHsd17β3	GGGCCTGGTTTGCCTGGTGAA	GCCATCGCCTGCTCCGGTAAT

### 2.4 Quantification of steroid production in culture medium

Culture medium was collected at each time point during harvest and stored at -80 °C until assayed. Progesterone (P4) and testosterone (T) levels in culture medium were quantified using the appropriate Cayman Chemical ELISA kits (Cat. # 582601 for P4; Cat. # 582701 for T) following the manufacturer’s instructions. Absorbance was measured using the Tecan NanoQuant Infinite M200 PRO microplate reader (Morrisville, NC). A standard curve was included in every plate to generate equations for the calculation of sample concentrations, and each sample (n=3–4 independent biological replicates) was assayed in duplicate. The total production of steroids (P4 and T) calculated from each biological sample was then divided by the number of viable cells (million) from the same T-25 flask.

### 2.5 Flow cytometry analysis of cell cycle phase distribution

To gain insights into the cellular processes underlying cell viability, cell cycle progression was analyzed by flow cytometry using propidium iodide staining. I-10 cells were treated with either DMSO, Cur 30, ATZ 33, ATZ 100, Cur 3 + ATZ 33, or Cur 3 + ATZ 100 µM (n=2 flasks/treatment/time point) as described in Methods, *2.2. Culture of Leydig cells and treatment*. At each time point (i.e., days 2, 4, and 6), treated cells were collected and rinsed with HBSS free of Ca^2+^ and Mg^2+^ (Cat. # SH30588.02, HyClone). Cells were fixed in cold 70% ethanol and stored at -20 °C until analysis. Following cell fixation, samples were washed with HBSS supplemented with 3% Fetal Bovine Serum, resuspended in propidium iodide staining buffer, and incubated with RNase A to digest RNA. After a 45-minute incubation at 37 °C, samples were stained with propidium iodide at a final concentration of 0.1 mg/mL and incubated for 1.5 hours at 37 °C. Fluorescence was measured using the BD Accuri C6 flow cytometer (BD Biosciences). The data were analyzed according to the Watson model (33) in FlowJo v10.10.1 (BD Biosciences).

### 2.6 Predicting binding affinity using AutoDock Vina

The 3D structures of ligands (i.e., chemicals of interest), Cur, ATZ, or endogenous substrates (cholesterol, pregnenolone, and androstenedione) for selected steroidogenic proteins, were downloaded from PubChem and saved as PDB files using ChimeraX 1.11.1 (34). For mouse steroidogenic proteins (StAR, Cyp11a1, Hsd3β6, and Hsd17β3), since there are no experimentally determined structures available from the RCSB Protein Data Bank (PDB) database, AI-generated structures were downloaded from the AlphaFold Protein Structure Database (https://alphafold.com/). For human steroidogenic proteins (StAR, CYP11A1, HSD3β2, and HSD17β3), experimental structures were obtained from the PDB database when available; otherwise, AlphaFold structures were used. Ligand and protein preparation, consisting of the addition of Gasteiger charges, hydrogens, and rotatable bonds, was carried out using “prepare_ligand” and “prepare_receptor” commands from the ADFR software suite (https://ccsb.scripps.edu/adfr/). In AutoDock MGL Tools v1.5.7 (https://ccsb.scripps.edu/mgltools/), a grid box size of 40 by 40 by 40 points (with adjusted exhaustiveness of 100 to account for the increased grid box size) and a default spacing of 0.375 angstroms was created around the substrate-binding domain of the proteins. Docking was executed using the above ligand, protein, and grid box configuration files in AutoDock Vina v1.2.7 (35, 36). The affinity binding scores calculated by AutoDock Vina are based on the most favorable pose (minimal energy and maximal interactions) of the ligand and the protein within the specified grid box. According to consensus, a binding score of 0 to -3 kcal/mol indicates a weak affinity, -4 to -6 kcal/mol a moderate affinity, and -7 or lower kcal/mol a strong affinity (37, 38). Re-docking was performed to validate the molecular docking protocol for the steroidogenic protein (CYP11A1), for which a co-crystallized structure (with its endogenous ligand cholesterol) is available in the PDB database. After re-docking with AutoDock Vina v.1.2.7, the separated cholesterol was superimposed against the original cholesterol pose in the co-crystallized PDB structure. A root mean square deviation (RMSD) value of 1.739 Å was generated by AutoDock MGL Tools 1.5.7, which is within the generally acceptable range of ≤ 2 Å (35), validating the molecular docking protocol used in this study.

### 2.7 Statistical analysis

To determine statistical significance among treatment groups, GraphPad Prism was used to conduct a one-way ANOVA followed by a Tukey’s *post-hoc* test (Version 10.5.0, Boston, MA). ELISA data were assessed for normal distribution with the Shapiro-Wilk test using R version 4.5.1 for samples with n=3 or more independent biological replicates. P-values less than 0.05 are considered statistically significant and are designated with either asterisks for comparison to control or specific letters for comparison among treatment groups (see figure legends).

## 3 Results

### 3.1 Effect of atrazine on I-10 Leydig cells

**Figure 2:**
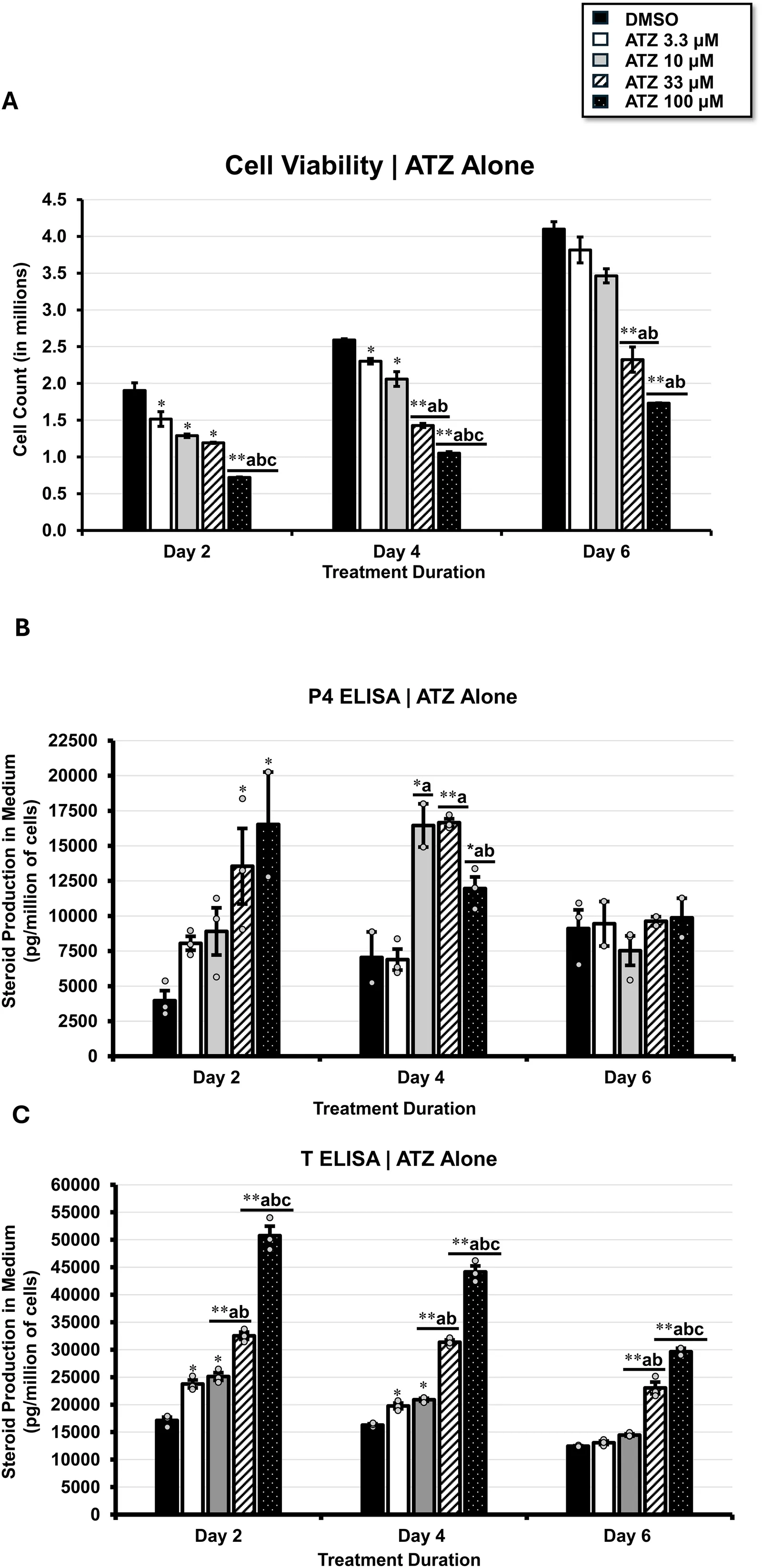
Effect of ATZ on cell viability and steroid production in I-10 cells. Effect of ATZ on I-10 cell viability **(A)**, P4 production **(B)**, and T production **(C)**. Bar graphs are represented as mean ± SEM (n=2–3 independent biological replicates). For Panels **(B, C)**, the values from the independent biological replicates are represented by the filled circles. Normal distribution of the data was confirmed with the Shapiro-Wilk test. Statistical significances are denoted with asterisks or letters: *p<0.05, **p<0.001 compared to DMSO; ^a^p<0.05 compared to ATZ 3.3 μM; ^b^p<0.05 compared to ATZ 10 μM; ^c^p<0.05 compared to ATZ 33 μM.

ATZ concentration-dependently decreased I-10 Leydig cell viability throughout the 6-day treatment (**Figure 2A**). This decrease in cell viability is witnessed even at the earliest time point, Day 2, with the lowest concentration, 3.3 µM. As anticipated, at the two higher concentrations (33 and 100 µM), ATZ decreased I-10 cell viability to an even greater extent (p<0.001) (**Figure 2A**, Day 2). Most notably, at 100 µM, cell viability was less than 50% of the control (DMSO), consistent across all time points.

Progesterone (P4) is an important steroidal hormone and is also a precursor of T in testicular steroidogenesis (**Figure 1**). Interestingly, despite causing a decrease in cell viability (**Figure 2A**), treatment with ATZ increased steroid production (calculated as P4 and T secreted into culture medium at the time of harvest per million viable cells). For P4 production, a significant but transient stimulation was observed on Day 2 at 33 and 100 µM, and on Day 4 at 10 and 33 µM, compared to control (**Figure 2B**). On the other hand, a clear concentration-dependent stimulation of testosterone (T) production is observed throughout the 6-day treatment (**Figure 2C**). The greatest stimulation of T production occurs at 33 and 100 µM (p<0.0001), across all time points.

**Figure 3:**
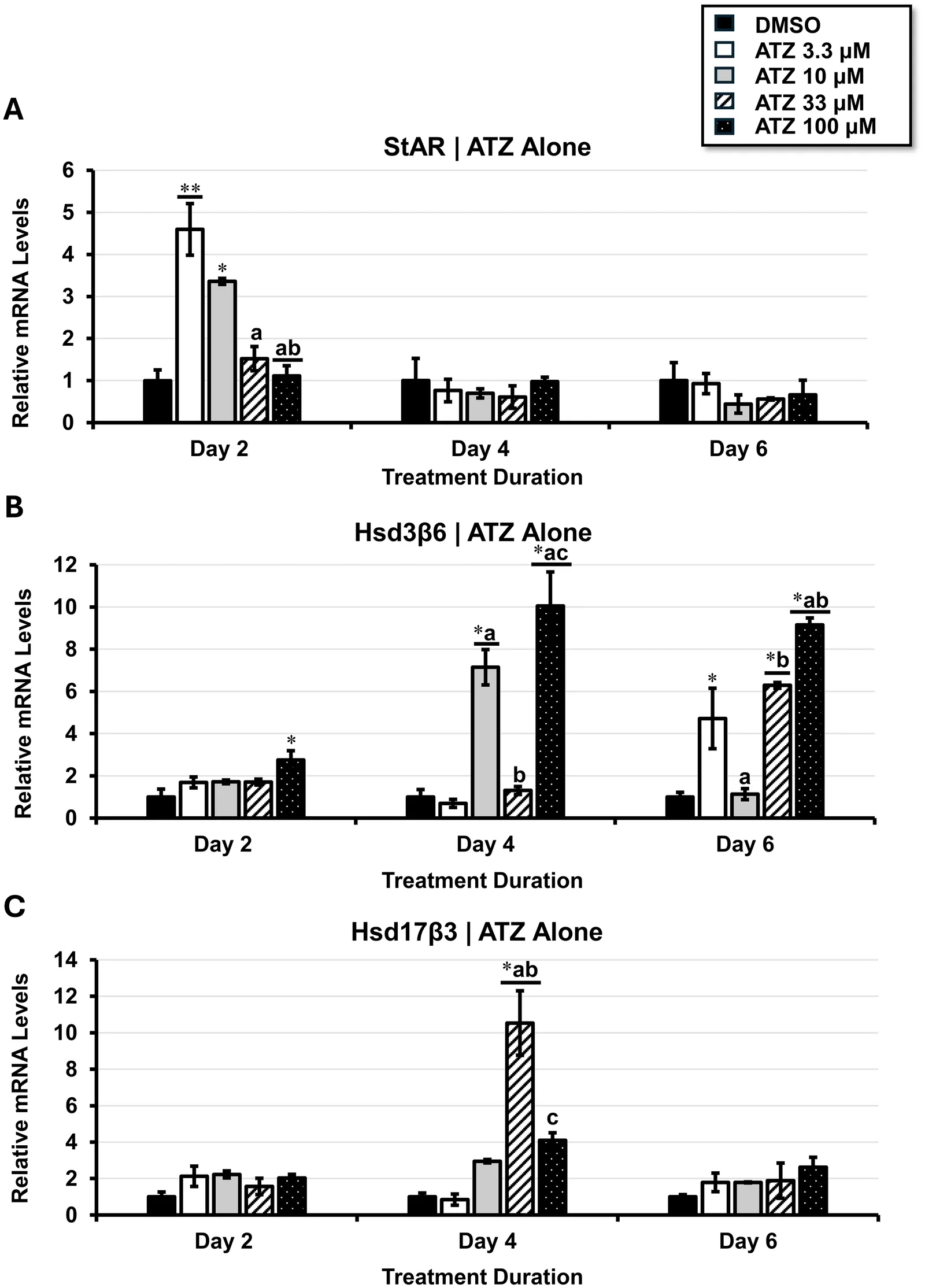
Effect of ATZ on the expression of steroidogenic genes in I-10 cells. **(A)** Effect of ATZ on StAR. The ratio between Day 2 DMSO and Day 4 DMSO is 138. The ratio between Day 2 DMSO and Day 6 DMSO is 58. Due to this, the DMSO for each time point is set as 1 (100%) and is used as a reference for the other treatment groups of the same time point. **(B)** Effect of ATZ on Hsd3β6. The ratio between Day 2 DMSO and Day 4 DMSO is 365. The ratio between Day 2 DMSO and Day 6 DMSO is 14303. Due to this, the DMSO for each time point is set as 1 (100%) and is used as a reference for the other treatment groups in the same time point. **(C)** Effect of ATZ on Hsd17β3. The ratio between Day 2 DMSO and Day 4 DMSO is 549. The ratio between Day 2 DMSO and Day 6 DMSO is 492. Due to this, the DMSO for each time point is set as 1 (100%) and is used as a reference for the other treatment groups in the same time point. Bar graphs are represented as mean ± SEM (n=2–3 independent biological replicates). Statistical significances are denoted with asterisks or letters: *p<0.05, **p<0.001 compared to DMSO; ^a^p<0.05 compared to ATZ 3.3 μM;^b^p<0.05 compared to ATZ 10 μM; ^c^p<0.05 compared to ATZ 33 μM.

To determine how ATZ affects the ability of I-10 cells to produce steroids, the expression of key steroidogenic factors, including a transporter and several enzymes, involved in the pathway of P4 and T biosynthesis, was assessed. Steroidogenic acute regulatory protein (StAR) is the protein involved in transporting cholesterol to the mitochondrial inner membrane (**Figure 1**). Consistent with the increased T production on Days 2 and 4 by ATZ 3.3 and 10 µM (**Figure 2C**), StAR showed a trend of early induction on Day 2 at 3.3 and 10 µM; however, this trend did not sustain at later time points (**Figure 3A**). Furthermore, StAR mRNA level in the control groups was highest on Day 2 but plummeted at later time points (i.e., ratio of day 2 to day 4 is 138; ratio of day 2 to day 6 is 58; see Figure Legends, **Figure 3A**), which is consistent with previous studies (39).

Hsd3β6 is the key enzyme required for the biosynthesis of P4 and Hsd17β3 is the key enzyme responsible for the conversion of androstenedione (the immediate precursor of T) to T (**Figure 1**). Correlating with the elevated P4 levels observed on Days 2 and 4 (**Figure 2B**) and the stimulation of T production (**Figure 2C**), inductions in the key steroidogenic enzymes Hsd3β6 and Hsd17β3 by ATZ 33 and 100 µM were observed. Hsd3β6 was significantly and consistently induced by ATZ 100 µM across all time points, including a 10-fold increase on Day 4 compared to control (**Figure 3B**). Besides 100 µM, at 33 µM, ATZ significantly induced Hsd3β6 on Day 6 (**Figure 3B**) and Hsd17β3 on Day 4 with a 10.5-fold increase compared to control (**Figure 3C**).

The two higher ATZ concentrations, 33 and 100 µM, were chosen for subsequent co-treatment studies with Cur because they decreased cell viability, increased steroid production, and induced key steroidogenic enzymes at specific time points.

### 3.2 Effect of curcumin on I-10 Leydig cells

**Figure 4:**
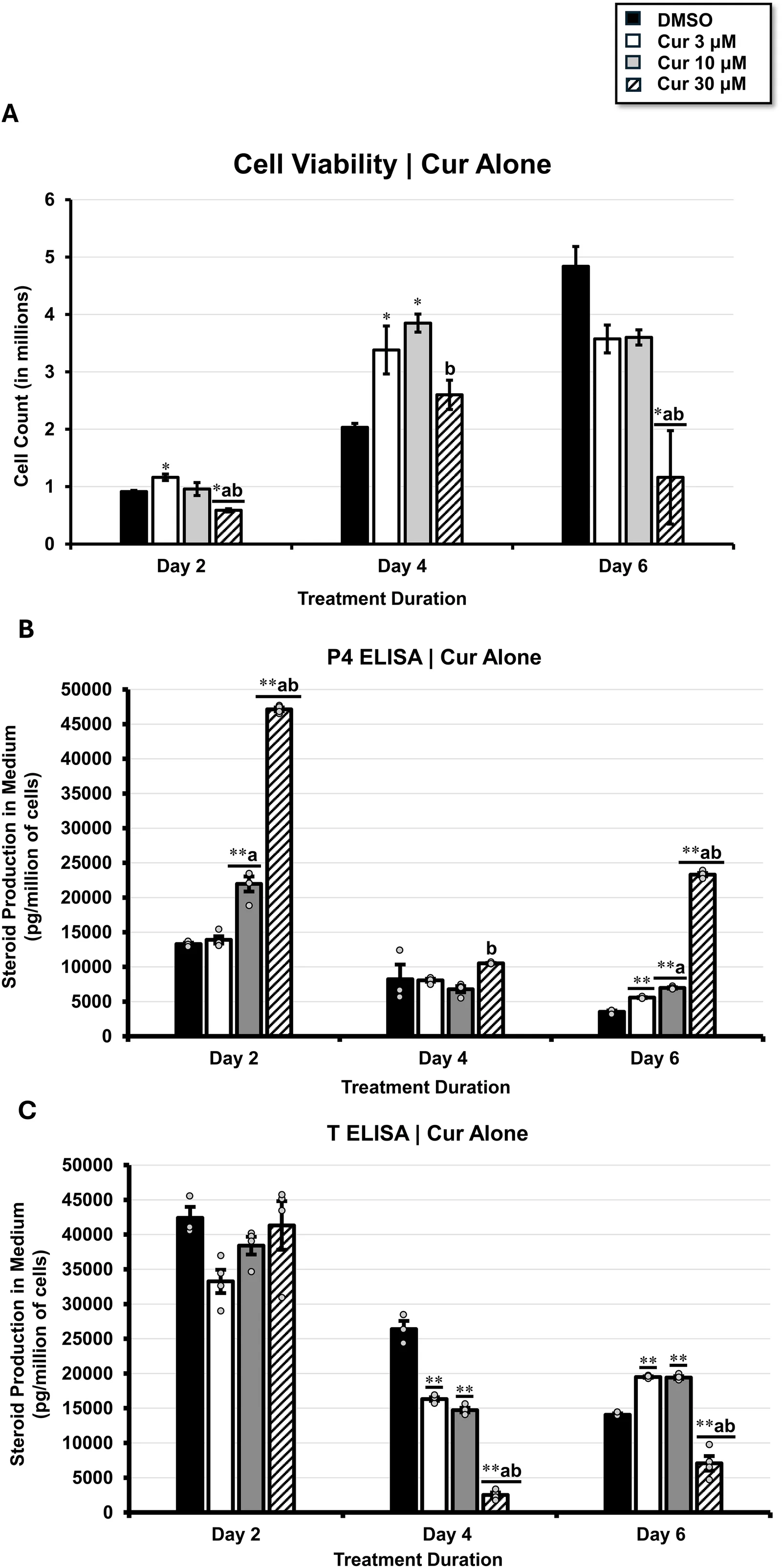
Effect of Cur on cell viability and steroid production in I-10 cells. Effect of Cur on I-10 cell viability **(A)**, P4 production **(B)**, and T production **(C)**. Bar graphs are represented as mean ± SEM (n=3–4 independent biological replicates). For Panels **(B, C)**, the values from the independent biological replicates are represented by the filled circles. Normal distribution of the data was confirmed with the Shapiro-Wilk test. Statistical significances are denoted with asterisks or letters: *p<0.05, **p<0.001 compared to DMSO; ^a^p<0.05 compared to Cur 3 μM; ^b^p<0.05 compared to Cur 10 μM.

Starting from Day 2, Cur 3 µM stimulated cell proliferation (**Figure 4A**). On Day 4, Cur 3 and 10 µM also significantly increased cell viability. At the highest concentration, 30 µM, cell viability declined on Day 2, and most notably on Day 6, showing a significant decrease compared to control and all lower concentration treatments (**Figure 4A**).

Regarding its impact on steroidogenesis, Cur treatment strongly and persistently stimulated P4 levels, most notably at the highest concentration (30 µM) across all time points (**Figure 4B**). On Day 2, P4 production was significantly stimulated by Cur 10 and 30 µM (**Figure 4B**). Cur at the lowest concentration, 3 µM, was able to stimulate P4 production at later time points (Days 4 and 6). On Day 6, Cur concentration-dependently increased P4 production to an even greater extent compared to Day 2 (**Figure 4B**).

Unlike the stimulation of T production by ATZ (at higher concentrations) in the face of reduced cell viability (**Figures 2A, C**), Cur exhibited more consistent signs of cytotoxicity compared to the ATZ-alone treatment, where T production was reduced with decreased cell viability. As shown in **Figures 4A, C**, Cur 30 μM decreased cell viability and reduced T production. T levels were only mildly stimulated on Day 6, at the lower concentrations of Cur, 3 and 10 µM (**Figure 4C**).

**Figure 5:**
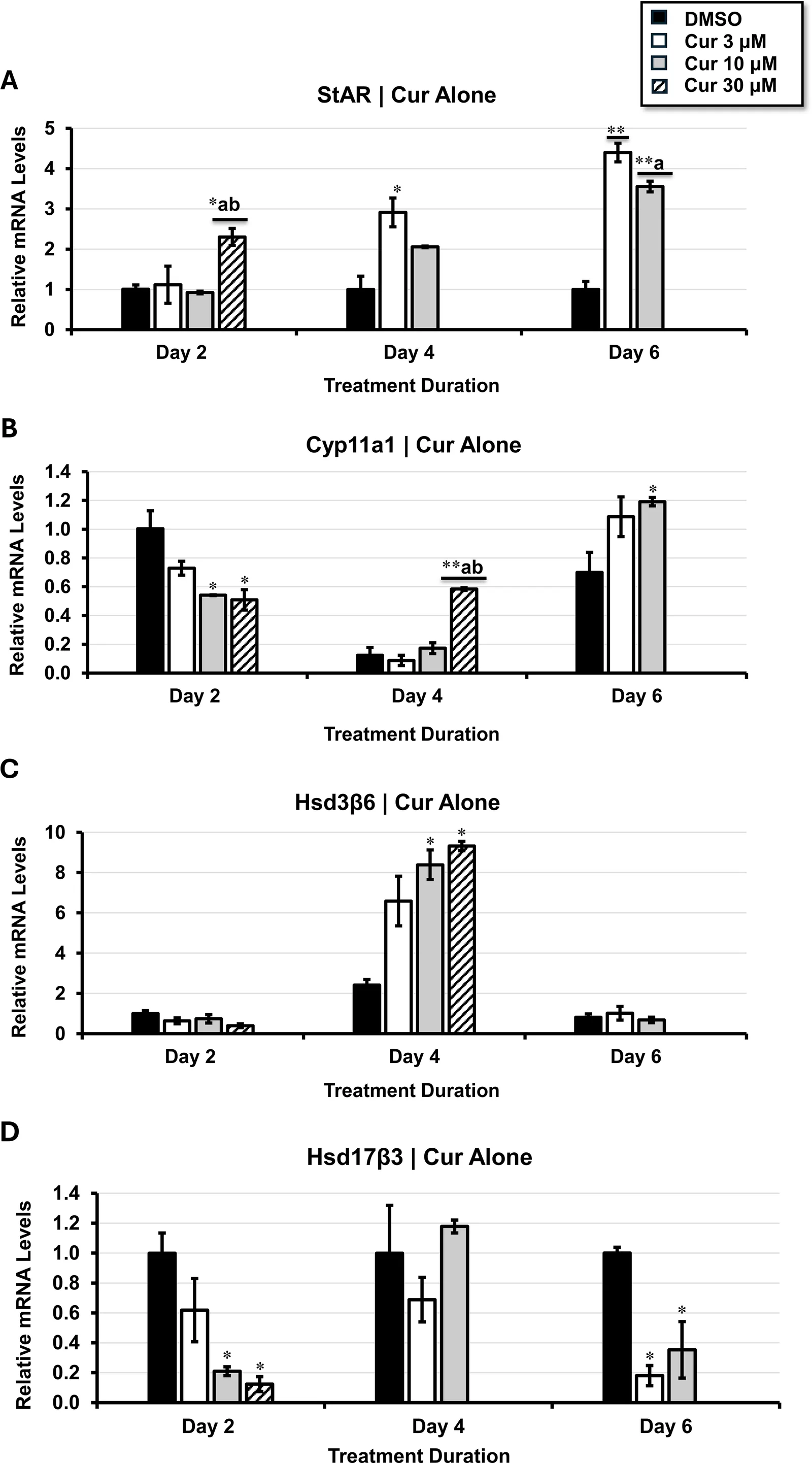
Effect of Cur on the expression of steroidogenic genes in I-10 cells. **(A)** Effect of Cur on StAR. The ratio between Day 2 DMSO and Day 4 DMSO is 59.18. The ratio between Day 2 DMSO and Day 6 DMSO is 18.70. Due to this, the DMSO for each time point is set as 1 (100%) and is used as a reference for the other treatment groups of the same time point. **(B)** Effect of Cur on Cyp11a1. Day 2 DMSO is set as 1 (100%) and is used as a reference for all other treatment groups throughout the 6-day treatment. **(C)** Effect of Cur on Hsd3β6. Day 2 DMSO is set as 1 (100%) and is used as a reference for all other treatment groups throughout the 6-day treatment. **(D)** Effect of Cur on Hsd17β3. The ratio between Day 2 DMSO and Day 4 DMSO is 17.52. The ratio between Day 2 DMSO and Day 6 DMSO is 0.05. Due to this, the DMSO for each time point is set as 1 (100%) and is used as a reference for the other treatment groups in the same time point. Bar graphs are represented as mean ± SEM (n=2–4 independent biological replicates). Statistical significances are denoted with asterisks or letters: *p<0.05, **p<0.001 compared to DMSO; ^a^p<0.05 compared to Cur 3 μM; ^b^p<0.05 compared to Cur 10 μM.

The gene expression results correlate, to some extent, with the steroid production, indicating that Cur was more likely to stimulate selected steroidogenic genes involved in P4 but not T production (**Figure 1**). The increase in P4 production at Cur 30 µM (**Figure 4B**) correlates with the transient induction of StAR on Day 2 in the same treatment (**Figure 5A**). Consistently, StAR mRNA level in the control groups was highest on Day 2 but plummeted at later time points (i.e., ratio of day 2 to day 4 is 59; ratio of day 2 to day 6: 19; see Figure Legends, **Figure 5A**). Therefore, although StAR mRNA levels were induced by Cur 3 µM on Day 4 and by Cur on Day 6 (**Figure 5A**), the absolute level of StAR mRNA on Day 6 was still less than Day 2 DMSO group, consistent with previous results (see Section 3.1).

The expression of Cyp11a1, the enzyme required for the conversion of cholesterol to pregnenolone (**Figure 1**), initially showed a trend of decrease on Day 2 with increasing concentrations of Cur, particularly for Cur at 10 and 30 µM (**Figure 5B**). In contrast, on Days 4 and 6, the opposite trend was seen. Specifically, Cyp11a1 transcript levels were significantly induced by Cur 30 µM on Day 4 and by 10 µM on Day 6 (**Figure 5B**). Additionally, corroborating with the observed stimulation of P4 production on Day 6 (**Figure 4B**), all concentrations of Cur (3, 10, and 30 µM) increased Hsd3β6 expression on Day 4 (**Figure 5C**).

Consistent with decreased T production on Day 4 and an overall lack of stimulation on T levels (**Figure 4C**), Cur did not stimulate the expression of Hsd17β3, but instead significantly down-regulated it on Day 2 by Cur 10 and 30 µM and on Day 6 by Cur 3 and 10 µM (**Figure 5D**).

The lowest concentration of Cur, 3 µM, was chosen for subsequent co-treatment studies because it increased cell viability early on, stimulated P4 (Days 4 and 6) and T (Day 6) production, and induced StAR expression at later time points (Days 4 and 6).

### 3.3 Effect of ATZ and Cur co-treatment on I-10 Leydig cells

To investigate the potential interaction between Cur and ATZ, two co-treatment groups were used: Cur at 3 µM with ATZ at 33 or 100 µM (i.e., Cur + ATZ 33 µM and Cur + ATZ 100 µM), along with individual treatments as controls.

**Figure 6:**
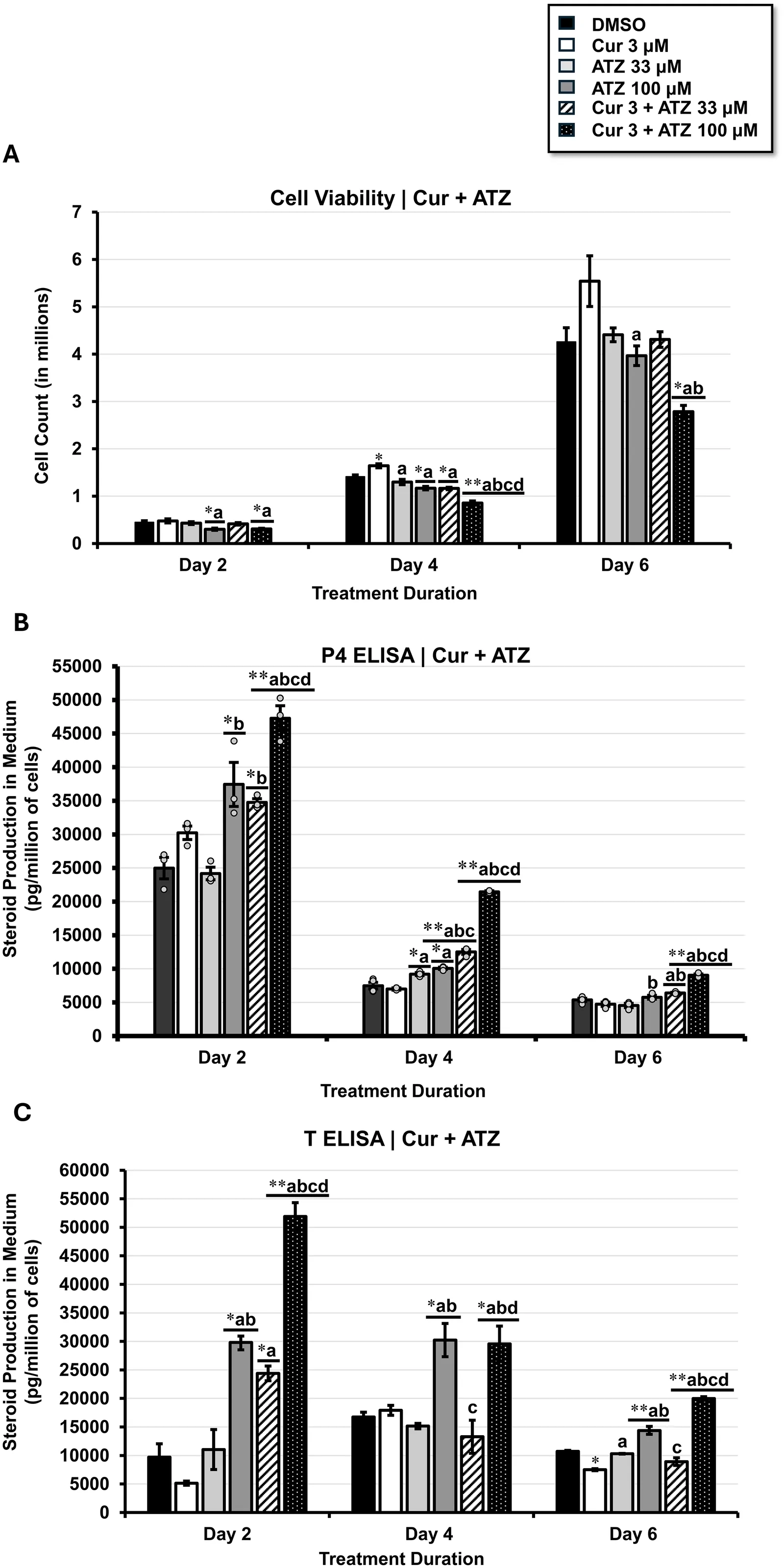
Effect of Cur + ATZ co-treatment on cell viability and steroid production in I-10 cells. Effect of Cur + ATZ co-treatment on I-10 cell viability **(A)**, P4 production **(B)**, and T production **(C)**. Bar graphs are represented as mean ± SEM (n=2–4 independent biological replicates). For Panels **(B, C)**, the values from the independent biological replicates are represented by the filled circles. Normal distribution of the data was confirmed with the Shapiro-Wilk test. Statistical significances are denoted with asterisks or letters: *p<0.05, **p<0.001 compared to DMSO; ^a^p<0.05 compared to Cur 3 μM; ^b^p<0.05 compared to ATZ 33 μM; ^c^p<0.05 compared to ATZ 100 μM; ^d^p<0.05 compared to Cur + ATZ 33 μM.

Cur alone showed a trend of stimulatory effect on cell viability at 3 µM on Day 4 (**Figures 4A**, **6A**), but no stimulation on cell viability was observed in the co-treatment groups; on the contrary, a clear trend of decreased cell viability was observed in the co-treatment groups, especially Cur + ATZ 100 µM, throughout the 6-day period (**Figure 6A**). Co-treatment of Cur + ATZ 100 µM decreased cell viability, which is consistently significant compared to Cur alone and control at all time points (**Figure 6A**). On Day 4, co-treatment of Cur and ATZ similarly decreased cell viability even with the co-treatment with the lower ATZ concentration, Cur + ATZ 33 µM (**Figure 6A**).

The addition of Cur significantly enhanced P4 and T production in the presence of ATZ 100 µM at all time points. The co-treatment with lower ATZ concentration (i.e., Cur + ATZ 33 µM) also increased P4 production compared to control and ATZ 33 µM on Day 2, and the co-treatment with higher ATZ concentration (i.e., Cur + ATZ 100 µM) stimulated P4 production compared to all other treatment groups at the same time point. On Day 4, P4 production increased by Cur + ATZ 33 compared to control and either single treatment group (i.e., Cur 3 or ATZ 33 µM) (**Figure 6B**). Cur + ATZ 100 µM showed consistent stimulation on P4 production (p<0.0001) at all time points (**Figure 6B**). A similar trend was also observed for T production, where the addition of Cur to ATZ 100 µM resulted in significant stimulation of T production compared to control and ATZ-alone treatments on Days 2 and 6 (**Figure 6C**). Therefore, the co-treatment regimens showed a clear trend of stimulation in both P4 and T levels. A summary of the effects of the single and co-treatments is presented in **Table 3**.

**Table 3:** Generalized trends of treatment effect on cell viability and steroid (P4 and T) production.

Treatment	Cell viability	P4 production	T production
ATZ	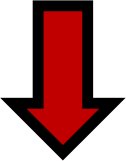	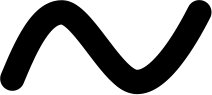	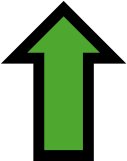
Cur	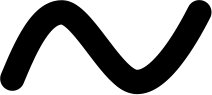	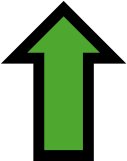	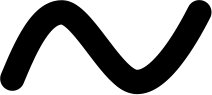
Cur + ATZ	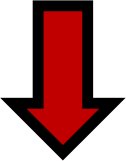	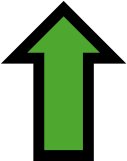	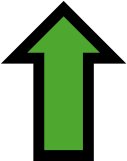

The upward and downward arrows represent increase and decrease, respectively, and the “~” represents no clear or persistent changes.

**Figure 7:**
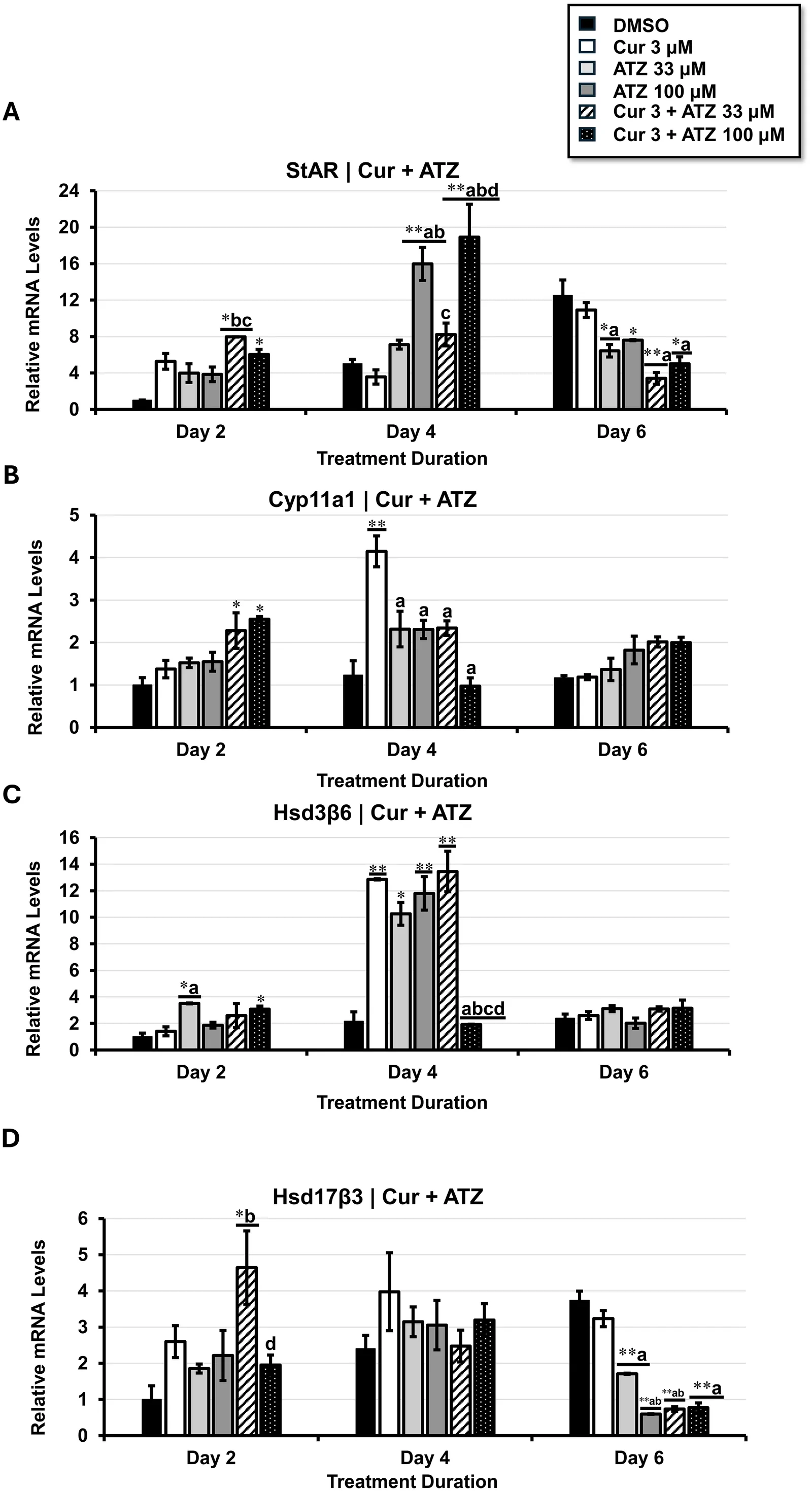
Effect of Cur + ATZ on the expression of steroidogenic genes in I-10 cells. Effect of Cur + ATZ on StAR **(A)**, on Cyp11a1 **(B)**, on Hsd3β6 **(C)** and on Hsd17β3 **(D)**. For all genes, Day 2 DMSO is set as 1 (100%) and is used as a reference for all other treatment groups throughout the 6-day treatment. Bar graphs are represented as mean ± SEM (n=2–4 independent biological replicates). Statistical significances are denoted with asterisks or letters: *p<0.05, **p<0.001 compared to DMSO; ^a^p<0.05 compared to Cur 3 μM; ^b^p<0.05 compared to ATZ 33 μM; ^c^p<0.05 compared to ATZ 100 μM; ^d^p<0.05 compared to Cur + ATZ 33 μM.

Next, we determined the impact of co-treatment on the expression of steroidogenic genes. The gene expression profile from the co-treatment groups correlated with steroid production to some degree, particularly at the earlier time points. Consistent with the P4 stimulation on Days 2 and 4 (**Figure 6B**), StAR, Cyp11a1, and Hsd3β6 were induced at specific time points. The increase in T level on Day 2 by Cur + ATZ 33 µM (**Figure 6C**) also correlated with an induction in Hsd17β3 at the same time point and concentration. On Day 2, both co-treatment groups significantly induced StAR expression, and a more robust stimulation was seen on Day 4 by Cur + ATZ 100 µM (**Figure 7A**). Both co-treatment groups also significantly increased Cyp11a1 expression on Day 2 (**Figure 7B**). Hsd3β6 was induced by Cur + ATZ 100 µM on Day 2 and Cur + ATZ 33 µM on Day 4, with a 6.2-fold increase compared to control of the same time point (**Figure 7B**). For Hsd17β3, only Cur + ATZ 33 µM induced its expression significantly compared to the control on Day 2 (**Figure 7B**). The observed changes in gene expression combined with the enhanced steroid production suggest a potential interplay between ATZ and Cur that further enhanced the steroidogenic process in I-10 cells.

**Figure 8:**
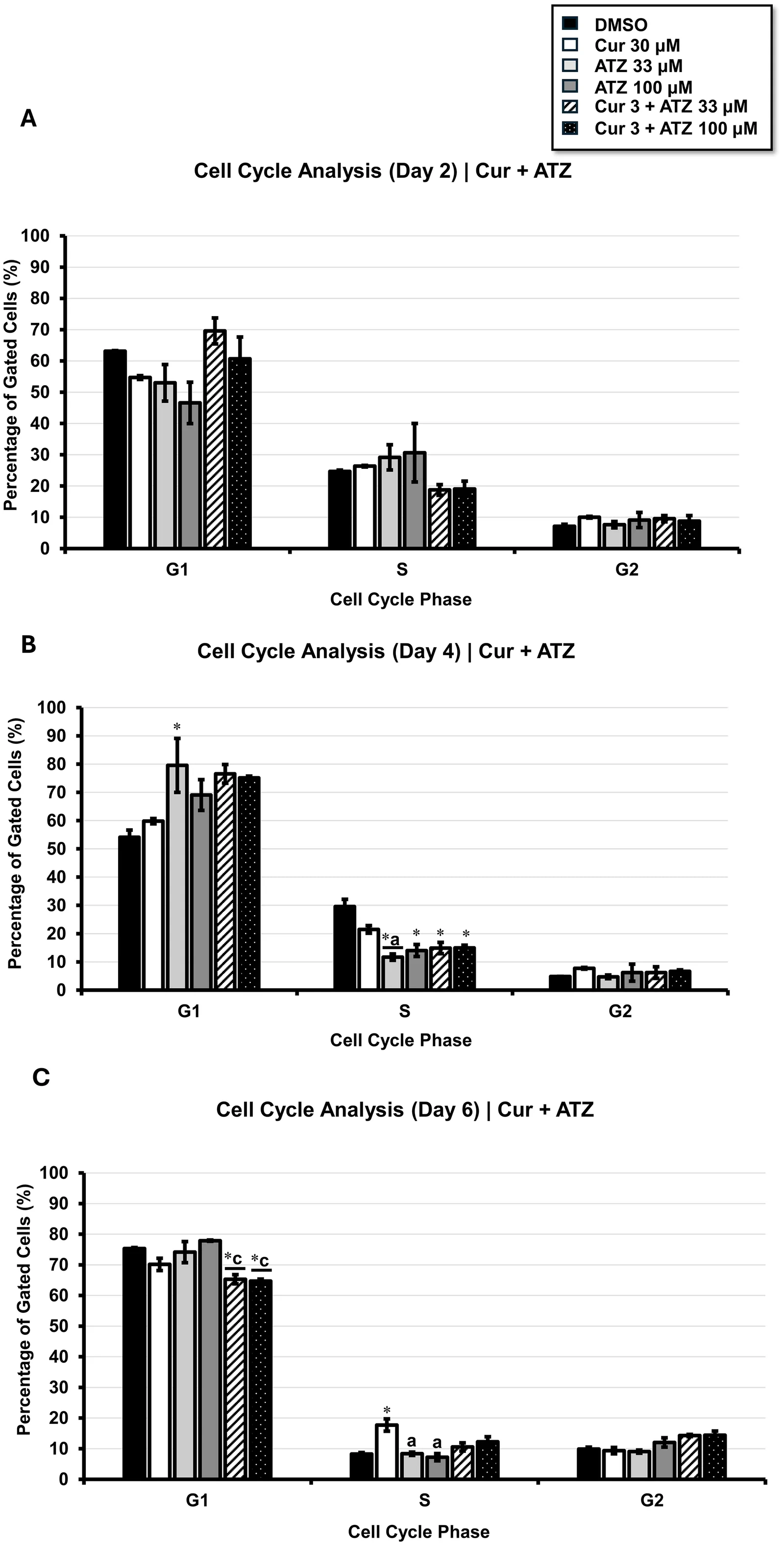
Effects of Cur, ATZ, and Cur + ATZ on cell cycle phase distribution. Effects of Cur, ATZ, Cur + ATZ on Day 2 **(A)**, Day 4 **(B)**, and Day 6 **(C)**. Bar graphs are represented as mean ± SEM (n=2 independent biological replicates). Statistical significances are denoted with asterisks or letters: *p<0.05, **p<0.001 compared to DMSO; ^a^p<0.05 compared to Cur 3 μM; ^b^p<0.05 compared to ATZ 33 μM; ^c^p<0.05 compared to ATZ 100 μM; d, p<0.05 compared to Cur + ATZ 33 μM.

The reduced cell viability observed correlated, to some degree, with changes in the cell cycle progression. ATZ treatments (particularly at higher concentrations such as 33 and 100 µM) consistently decreased cell counts throughout the 6-day treatment (**Figure 2A**), which correlates with the significant increase in the percentage of cells in the G1 phase and the concomitant decrease in cells in the S phase on Day 4 (**Figure 8B**), indicating a cell cycle arrest at the G1 phase. Curcumin at the highest concentration (30 µM) showed a marked growth inhibition on Day 6 (**Figure 4A**) and consistently, cells from the same treatment had a significant greater S phase percentage but similar G1 and G2 percentages compared to control (**Figure 8C**), suggesting S phase arrest. In the co-treatment study on Day 4, all treatment groups containing ATZ reduced viability (**Figure 6A**), consistent with the reduction in the S phase percentage in the same treatments on the same time point (**Figure 8B**, S phase), indicating that ATZ reduces the capability of I-10 cells to proliferate, regardless of the presence of Cur. Furthermore, in both co-treatment groups, significantly fewer cells entered the G1 phase on Day 6, compared to control and ATZ alone treatments (**Figure 8C**).

### 3.4 Predicted binding affinities of ATZ and Cur to key steroidogenic proteins

Molecular docking of the ligand (ATZ or Cur) and the target protein (i.e., selected steroidogenic factors) was performed to predict the strength of direct binding. When ATZ was docked against key steroidogenic proteins (StAR, Cyp11a1, Hsd3β6, and Hsd17β3), its binding affinity scores suggested moderate binding strength for all steroidogenic proteins, both mouse and human origins, weaker than that of Cur or the endogenous substrates (**Tables 4**–**7**). Unlike ATZ, when Cur was docked against the same set of steroidogenic proteins, strong binding affinity scores were obtained that are comparable to the endogenous substrates for all the steroidogenic proteins (**Tables 4**–**7**), indicating a high likelihood of interactions between Cur and the key steroidogenic factors tested.

**Table 4:** Predicted binding affinities of endogenous substrates to mouse steroidogenic proteins.

Ligand	Target protein	Alphafold ID	Binding affinity (kcal/mol)	Binding strength
Cholesterol	mStAR	AF-P51557-F1	**-9.301**	**Strong**
Cholesterol	mCyp11a1	AF-Q9QZ82-F1	**-9.138**	**Strong**
Pregnenolone	mHsd3β6	AF-O35469-F1	**-9.353**	**Strong**
Androstenedione	mHsd17β3	AF-P70385-F1	**-8.25**	**Strong**

Ligands with strong binding affinity to the target protein are bolded in the tables.

**Table 5:** Predicted binding affinities of ATZ and Cur to mouse steroidogenic proteins.

Ligand	Target protein	Alphafold ID	Binding affinity (kcal/mol)	Binding strength
ATZ	mStAR	AF-P51557-F1	-5.659	Moderate
ATZ	mCyp11a1	AF-Q9QZ82-F1	-5.867	Moderate
ATZ	mHsd3β6	AF-O35469-F1	-6.189	Moderate
ATZ	mHsd17β3	AF-P70385-F1	-6.454	Moderate
**Cur**	mStAR	AF-P51557-F1	**-8.621**	**Strong**
**Cur**	mCyp11a1	AF-Q9QZ82-F1	**-8.269**	**Strong**
**Cur**	mHsd3β6	AF-O35469-F1	**-9.115**	**Strong**
**Cur**	mHsd17β3	AF-P70385-F1	**-9.225**	**Strong**

Ligands with strong binding affinity to the target protein are bolded in the tables.

**Table 6:** Predicted binding affinities of endogenous substrates to human steroidogenic proteins.

Ligand	Target protein	PDB/alphafold (AF) ID	Binding affinity (kcal/mol)	Binding strength
Cholesterol	StAR	pdb_00003p0l	**-8.739**	**Strong**
Cholesterol	CYP11A1	pdb_00003n9y	**-11.457**	**Strong**
Pregnenolone	HSD3β2	AF-P26439-F1	**-8.45**	**Strong**
Androstenedione	HSD17β3	AF-P37058-F1	**-9.039**	**Strong**

Ligands with strong binding affinity to the target protein are bolded in the tables.

The differences in binding scores between ATZ and Cur can be attributed to the differences in their structural and physical properties. The binding affinity score calculated by AutoDock Vina reflects the overall likelihood of intermolecular interactions; the more negative the score, the greater the likelihood (35). Cur is predicted to have more negative binding affinity scores compared to ATZ due to more possible favorable intermolecular interactions. As shown in **Tables 5**, **7**, Cur is predicted to have a greater number of interactions with the protein target through: (1) pi interactions from its two terminal aromatic rings (See Cur structure in **Table 1**), (2) a greater capacity for dipole interactions as indicated by its increased polar surface area, and (3) the potential for more hydrogen bonding, allowing for more favorable binding compared to ATZ.

**Table 7:** Predicted binding affinities of ATZ and Cur to human steroidogenic proteins.

Ligand	Target protein	PDB/alphafold (AF) ID	Binding affinity (kcal/mol)	Binding strength
ATZ	StAR	pdb_00003p0l	-5.836	Moderate
ATZ	CYP11A1	pdb_00003n9y	-6.219	Moderate
ATZ	HSD3β2	AF-P26439-F1	-6.346	Moderate
ATZ	HSD17β3	AF-P37058-F1	-6.436	Moderate
**Cur**	StAR	pdb_00003p0l	**-8.673**	**Strong**
**Cur**	CYP11A1	pdb_00003n9y	**-9.618**	**Strong**
**Cur**	HSD3β2	AF-P26439-F1	**-9.32**	**Strong**
**Cur**	HSD17β3	AF-P37058-F1	**-9.222**	**Strong**

**Tables 4**–**7** Experimentally determined protein structures were obtained from the RCSB PDB database when available. If not, AI-predicted structures were obtained from AlphaFold. Docking scores were calculated using AutoDock Vina v1.2.7. For the human StAR protein (**Tables 6**, **7**), Chain B was used for docking analyses. Hsd3β2 is the human orthologue of the mouse Hsd3β6 protein. Re-docking was performed on the co-crystallized human CYP11A1 protein with its endogenous ligand cholesterol, yielding a RMSD value of 1.739 Å (within the acceptable range of ≤ 2 Å). Binding strength categories are based on commonly reported Vina score ranges. A binding score of 0 to -3 kcal/mol indicates weak strength, -4 to -6 kcal/mol moderate strength, and -7 or lower kcal/mol as strong (37, 38). Ligands with strong binding affinity to the target protein are bolded in the tables.

## 4 Discussion

In the present study, we quantified the amount of steroids (P4 and T) secreted by I-10 cells into the culture medium as the readout of total steroid production, which was then divided by the number of viable cells (million) from the same T-25 flask. This method took into consideration the variations in seeding density, overall viability associated with the respective exposure in each T-25 flask, and the ability of Leydig cells to produce steroids after 48 hours of specified exposure. For ATZ treatment, T production significantly and consistently increased throughout the 6-day treatment despite gradually decreasing viability, indicating that T production was somehow cranked up even in the presence of ATZ-induced cytotoxicity. This is consistent with previous *in vitro* and *in vivo* studies (40, 41) and may be attributed to a stress-induced response. This assumption is supported by *in vivo* studies, which reported the stimulation of T as transient because, over time, it became apparent that steroid production by the testes eventually decreased with increased exposure to ATZ (42). Not surprisingly, Cur at a high concentration (30 µM) shows toxicity similar to ATZ exposure, indicated by a decrease in cell viability (**Figure 4A**) on Days 2 and 6, accompanied by P4 stimulation on the same time points (**Figure 4B**). Consistent with the observed cell growth inhibition, ATZ (30 and 100 µM) caused G1 arrest and associated S phase reduction on Day 4 (**Figure 8B**), while Cur (30 µM) did not cause G1 arrest on Day 4 but caused an increase in cells in the S phase on Day 6 (**Figures 8B, C**). ATZ maintained a trend of G1 arrest on Day 6 while Cur increased the S phase percentage (**Figures 8B, C**), suggesting that ATZ and Cur may reduce cell proliferation capacity through distinct mechanisms.

Regarding steroidogenesis, it is noteworthy that the stimulation by Cur on P4 levels did not result in increased T production (**Figure 4C**), which may be due to the reduced expression of Hsd17β3 under Cur exposure, which is the enzyme that catalyzes the last reaction in T biosynthesis (**Figure 1** and **Figure 5D**). On the other hand, ATZ-induced increases in P4 (**Figure 2B**) did correlate to increased T levels (**Figure 2C**), indicating that ATZ may tap into some regulatory mechanism to ramp up the basal steroid production in Leydig cells at the expense of decreased cell viability. If this abnormal stimulation of T production by ATZ, which is likely to be transient, can be prevented through crosstalk with Cur, then perhaps we may be able to prevent the eventual decline of steroid production and exhaustion of Leydig cells observed in previous *in vivo* studies (42), and thus preserve Leydig cells’ steroidogenic capacity in a more sustainable manner.

When I-10 cells were treated with both Cur and ATZ, cell viability was generally comparable to the respective ATZ alone treatment and significantly lower than the control group (**Figure 6A**). On Day 4, the viability data correlate with cell cycle distribution in that decreased viable cell count is corroborated by a trend in G1 arrest and decreased S phase percentage (**Figures 6A**, **8B**). This indicates that Cur could not restore cell viability in the presence of ATZ, despite the growth-stimulatory effects seen in Cur alone treatment on Days 2 and 4 (**Figure 4A**). Interestingly, the decrease in viability in co-treatment is accompanied by stimulation of P4 production (**Figure 6B**). Co-treatment also significantly increased T production, compared to control (DMSO) and either single treatment (**Figure 6C**), despite decreased Hsd17β3 expression observed on Day 6 (**Figure 7D**). For the co-treatment with higher ATZ concentration (Cur + ATZ 100 µM), the stimulation on production of P4 and T was always more prominent (**Figure 6**), which may be because the higher concentration of ATZ (100 µM) was more likely to elicit an effect with Cur than the lower concentration (33 µM). While the addition of Cur may not restore I-10 cell viability, it does seem able to mitigate the abnormal stimulation of T production by ATZ to some degree, especially with ATZ 33 µM.

Furthermore, molecular docking of Cur to mouse and human steroidogenic proteins revealed strong binding potential across species and correlated with the *in vitro* results of this study. The consistent cross-species strong binding predicted between Cur and key steroidogenic proteins by molecular docking suggests possible direct interaction (**Tables 5**, **7**), which may result in favorable transporter and/or enzymatic activities. Congruent with this speculation, at the earliest time point tested (i.e., Day 2), Cur stimulated P4 production, either alone (**Figure 4B**) or in the presence of ATZ at 100 µM (**Figure 6B**), suggesting that steroidogenic proteins involved in P4 biosynthesis may have been stimulated by Cur to achieve greater productivity even in the presence of ATZ, which is predicted to have weaker binding affinity to the same set of steroidogenic proteins (**Tables 5**, **7**). On the other hand, Cur exhibited the strongest binding with Hsd17β3 (**Tables 4**–**7**), even stronger than the endogenous substrate (androstenedione). Such strong binding, however, did not seem to have any major impact on T production on Day 2 (**Figure 4C**). It should be noted that the molecular docking experiments in this study did not account for hydrophobic effects from the displacement of water molecules in a solvated environment, the flexibility of amino acid residues in proteins, or different possible conformations of the target protein. Also, co-crystallized and experimentally derived protein structures were not available in the PDB database for most of the steroidogenic proteins relevant to our study, so molecular docking was carried out using protein structures from the AlphaFold database, predicted by AI. The major drawback of this approach is that it lacks experimental validation and can only offer predictions of the binding between the ligand and the protein. Improvement would come from future studies employing post-modeling refinement of AlphaFold protein structures, along with experimental validation, such as a cellular thermal shift assay to confirm binding strength (43, 44).

The wide range of concentrations used in this study (1, 3, 10, 30, 100 µM), while not directly relevant to environmentally relevant *in vivo* exposures, are useful in establishing points of departure (PoD) for ATZ and Cur effect to determine the lowest concentration at which adverse effects are first observed, in addition to revealing insights into the potential mechanisms on how T production in Leydig cells is modulated by the chemical (45, 46). The I-10 cell line used in this study is tumor-derived and may not fully recapitulate normal Leydig cell physiology. The use of primary Leydig cells may also be limited, as they eventually lose their ability to synthesize steroids after a few days of culture (47). In order to overcome the lack of physiological and translational relevance of the *in vitro* models, a longer-term animal study would be required to determine whether co-exposure of ATZ and Cur can indeed prevent or mitigate ATZ’s toxic effects on T production and/or preserve the adult Leydig cell population *in vivo*. Although we designed our study to achieve a longer exposure period (i.e., from typical 3 days to extended 6 days) and collected data for cell viability, steroid production, and gene expression profiles all from the same biological samples, ensuring high consistency, the results are limited because they were from a specific cell line and only indicate potential interplay between ATZ and Cur, but cannot shed light on which pathway(s) or target(s) Cur and/or ATZ act on to cause the changes witnessed in viability, steroidogenesis, and/or cell cycle profiles. Given the important role mitochondria play in steroidogenesis (**Figure 1**), it would be valuable for future studies to characterize their status by quantifying their amount and function. An additional measure to confirm the protein levels and/or enzymatic activities of the steroidogenic enzymes would also be worthwhile for future studies, as only relative mRNA levels are reported in this study. Quantification of mRNA changes is still valuable, allowing correlation of the expression profile of key steroidogenic factors with the production of individual steroids (P4 and T). Our results also suggest that Cur and ATZ do not apparently tap into the cyclic adenosine monophosphate (cAMP) signaling pathway, which is utilized by LH to stimulate steroidogenesis in Leydig cells. Because, if this were the case, StAR, as well as other steroidogenic genes, would have been more robustly and persistently induced (48), yet in the present study, there is no sustained elevation of StAR mRNA levels (**Figure 7A**).

Looking ahead, knowledge of the effects of Cur and ATZ on testicular steroidogenesis, along with an understanding of the mechanisms by which they act upon, can have significant clinical relevance. Such knowledge not only helps mitigate the endocrine-disrupting effects of ATZ but may also pave the way for novel applications to manage stressors for Leydig cell protection.

## Data Availability

The raw data supporting the conclusions of this article will be made available by the authors, without undue reservation.
